# Conventional and Alternative Strategies to Cope With the Subtropical Climate of Tokyo 2020: Impacts on Psychological Factors of Performance

**DOI:** 10.3389/fpsyg.2019.01279

**Published:** 2019-06-04

**Authors:** Guillaume R. Coudevylle, Stéphane Sinnapah, Nicolas Robin, Aurélie Collado, Olivier Hue

**Affiliations:** Laboratory ACTES (UPRES-EA 3596), University of the French West Indies, Pointe-à-Pitre, France

**Keywords:** cognitive abilities, hot-wet climate, heat stress, cooling, thermal comfort, mental technique

## Abstract

The thermal discomfort caused by a hot or hot-wet climate can have negative effects on human performance. The 2020 Summer Olympic and Paralympic Games will take place in Tokyo’s hot and humid summer period, possibly exposing athletes to severe environmental stressors. In addition to technical, tactical, physical and nutritional preparation, Olympians and Paralympians need an optimal psychological state to turn in their best performances, especially in terms of emotional control, concentration and motivation. Yet, the tropical climate can have many negative effects on these factors. Better understanding of the negative effects of this climate and the strategies to manage them might be crucial for competitors, coaches and their teams in Japan. At the psychological level, cooling interventions before, during and/or immediately after exercise were mainly studied on perceptual responses. However, the effects of these interventions on other psychological components such as cognitive abilities or psychological states and the use of psychological techniques have been little explored, especially in hot-wet climate. Thus, this article proposes to take stock of the knowledge on the conventional and alternative strategies that help athletes to psychologically cope with the subtropical climate of Tokyo.

## Introduction

Competing at the Olympic/Paralympic Games requires high level physical, technical, tactical, nutritional, and mental preparation. Athletes have to manage event stressors like anxiety (Nicholls and Levy, 2016) and competitive stressors like preparation, expectations, and opponents (Fletcher and Sarkar, 2012). Tokyo, lying in a humid subtropical zone, will add another important stressor: the hot and wet climate in the summer season. The Köppen Climate Classification subtype for this climate is warm temperature, fully humid and hot summer (i.e., subtropical climate). The hottest month of the year is August with an average temperature of 27.4°C (81.3°F) corresponding to the month of the Games. The average temperatures in Tokyo have risen by three degrees Celsius in the last one hundred years. Two degrees of these three are attributed to the urban heat island (UHI) effect and one to global warming. UHI is a kind of heat accumulation phenomenon in which temperatures in urban areas are markedly higher than those in surrounding areas (Matsumoto et al., 2017). One of the causes of this effect is asphalt concrete (i.e., the most common pavement surfacing materials) exposed to solar irradiation (Mohajerani et al., 2017). During the competitions, athletes from certain disciplines will be directly exposed (e.g., triathlon, road cycling, marathon, 20 and 50 km race walk). The particularities of Tokyo 2020 are required to mobilize all strategies to succeed in achieving sports performance in high thermal stresses without risk for health. Associated with intense physical activity, if not regulated, these high thermal stresses may provoke hyperthermia, which disrupts brain function (e.g., cerebral blood flow reduction, brain temperature increase, and cerebral oxygenation compromising), which in turn alters the cognitive abilities (Bain et al., 2015). Therefore, it is important to determine strategies that allow better regulation (e.g., cooling intervention) and better adaptation to this hyperthermia (e.g., acclimatization, mental preparation) without risk to health. In addition to the heat-related physiological problems that affect athletes’ health, notably heat stroke (Brotherhood, 2008), the thermal environment can have negative consequences on performance (Hue, 2011). During prolonged aerobic exercise, this stressor is known to negatively impact both physiological (e.g., Maughan et al., 2012) and psychological performances (e.g., Vasmatzidis et al., 2002). The literature proposes cooling strategies to deal with these deleterious effects of heat (Bongers et al., 2017). Regarding the psychological components, most studies are interested in the perceptual responses of heat stress with or without cooling intervention. However, the effects of these interventions on other psychological components such as cognitive abilities (e.g., reaction time, attention, executive function) or psychological states (i.e., mood, emotional, expectation, and motivational effects), and the use of psychological interventions, are little known, specifically in the tropical climate (i.e., TropC). This brief review deals with the available strategies to cope with the impact of hot and hot-wet climates on perceptual responses and cognitive abilities. We first focus on the effects of conventional strategies (i.e., training in the thermal conditions and cooling interventions). We then focus on alternative or complementary strategies (i.e., menthol ingestion techniques and mental techniques). We also aimed to study the impact of these different strategies on other psychological components that have not been examined so far (e.g., self-confidence, motivation, flow states). In addition to the theoretical interest, this article provides information to coaches on how best to prepare their athletes for the climate conditions of Tokyo 2020 but also for the upcoming events (e.g., 2022 FIFA World Cup Qatar). To establish this mini-review, citations from *Pubmed* and *Sciencedirect* were identified from the earliest record until April 2019 using the following search terms: hot climate, hot-wet climate, strategies, sport, performance, and psychology. Included studies required using adult participants (≥18 years) and ambient temperatures (≥35°C and ≤ 40% rH for studies conducted in hot climates and around 31°C and ≥ 50% rH for studies conducted in TropC). All studies without any link or involvement in competitive physical activities were excluded.

## Impacts of Conventional Strategies to Deal With Thermal Stress

The main usual strategies to limit the negative effects of heat or TropC are training in the thermal stress and cooling interventions (for a review, see Bongers et al., 2017). For greater clarity, we distinguished the studies conducted in hot climate from those conducted in TropC and distinguished the factors related to perceptual responses from those related to cognitive abilities.

### Training in Hot and Tropical Climate

#### In Hot Climate

Thermal adaptation encompasses physiological factors, behavioral adjustments, and psychological factors (Marialena and Koen, 2003). According to Pryor et al. (2019), heat acclimation in a hot environment is induced by repeated exercise-heat exposures that result in temporary physiological adaptations. This reduces thermal load and cardiovascular strain, and improves heat dissipation mechanisms during exercise. In addition, Taylor (2014) reviewed that the adaptations previously evoked can improve aerobic performance, enhance exercise-heat tolerance, and reduce the risk of exertional heat illness. Likewise, a series of studies investigated whether training in the same thermal conditions as the competition would enable athletes or soldiers to better adapt to heat stress (see Heathcote et al., 2018, for a review). Several studies showed an improved thermal comfort after acclimation (Sunderland et al., 2008; Costa et al., 2014). Recently, Malgoyre et al. (2018) compared the effects of a 15-day aerobic training program on soldiers in a hot-dry versus a temperate environment. They showed that the physiological modifications (e.g., rectal temperature) were mostly the same in the two environments but that thermal discomfort and the rating of perceived exertion (RPE) were much lower in the heat-training group than in the control group. Equivalent results were obtained for thermal comfort, sensation and perceived exertion in athletes prior to the Marathon des Sables following short-term heat acclimation (Willmott et al., 2017). Being accustomed to withstanding high training loads and high environmental stresses, athletes perceive the difficulty of an exercise as less onerous than the objective evaluation of it, especially compared to less well-accustomed athletes.

Several studies have shown the positive effects of thermal adaptation on cognitive abilities. Radakovic et al. (2007) examined the effects of exertional heat stress and acclimation status on the physiological and cognitive abilities of soldiers. The participants performed an exertional heat stress test in a cool environment, a hot environment to which they were not acclimatized, or a hot environment after undergoing 10 days of passive or active acclimation. The results showed that participants in the acclimatized group did not experience any adverse effects of heat stress, unlike the group of non-acclimatized participants who experienced a slight decrease in attention. Although different, these populations of athletes and soldiers may have commonalities in coping with a stressful environment (e.g., rigor, resilience, determination).

#### In Tropical Climate

The literature on the impact of acclimation to TropC on psychological factors is far sparser. Schmit et al. (2017), testing the impact of an 8-day training camp, with or without a cooling vest, on 13 triathletes who performed two 20-km cycling time-trials in TropC (35°C and 50% rH), found positive effects on thermal comfort. Then, it can be assumed that acclimation to the environment reduces the effects of heat stress and thus avoids the risk of cognitive disturbances. Thus, training in TropC may be one of the most important strategies to sensitize the delegations to Tokyo 2020. In this regard, Racinais et al. (2015) recommended repeated exercise-heat exposures over 1–2 weeks, as well as incentives for hydration during the athletes’ effort.

### Cooling Interventions

Cooling interventions, such as cold-water immersion/ingestion or cooling garments, have been developed to prevent the physiological and psychological consequences of heat and TropC (for a review, see Jones et al., 2012; Bongers et al., 2017).

#### In Hot Climate

Ruddock et al. (2017) reviewed the effectiveness of cooling strategies during continuous exercise in heat. The results were from studies in a hot climate with lower relative humidity, but they are nonetheless interesting and indicate that cooling decreases the RPE and thermal perception during fixed-intensity exercise, which would improve endurance performance. Several studies have also found that head cooling decreases the RPE and thermal discomfort during hyperthermic exercise (e.g., Armada-da-Silva et al., 2004; Mündel et al., 2007). For example, Simmons et al. (2008) tested nine physically active, non-heat-acclimated volunteers. Participants performed two exercises (i.e., 12-min constant-load cycling tests at 70% VO^2^max) separated by a 90-min period of passive heating in two conditions: with or without head and face cooled. They showed that head cooling during passive heating reduced RPE and improved thermal comfort during subsequent exercise in the heat.

While cooling interventions in the heat appear to have positive effects on the RPE and thermal comfort, the benefits to cognitive abilities are less clear-cut. Their effectiveness seems limited and dependent on the timing of implementation (Schmit et al., 2017) and the type of task (Shibasaki et al., 2017). Along this line, Gaoua et al. (2011) assessed whether attention and memory task performance would be positively affected by cold pack application to the head during passive heating compared to passive heating without cold packs and a control situation. Head cooling had a more positive effect on working memory capacity, and rapid visual processing was no longer negatively impacted, which was the case for passive heating without the cold packs. However, head cooling showed no beneficial effect on pattern recognition memory. In another experiment with three conditions (hot, 50°C vs. hot, 50°C with cold packs vs. control, 20°C), Racinais et al. (2008) investigated whether cooling (i.e., cold packs to the head) would limit the alterations in motor training and cognitive function induced by the passive hyperthermia. They showed that cooling preserved memory capacity but not visual memory.

#### In Tropical Climate

Arngrimsson et al. (2004) showed that wearing a cooling vest during warm-up in TropC reduces thermal discomfort at the start of a 5-km run race compared to control condition, but this decrease fades after 3.2-km of running. That said, according to the authors, reducing perceived thermal discomfort and cardiovascular strain at the beginning of the race seems to play a role in improving performance in TropC (i.e., 32°C and 50% rH). Moreover, Cleary et al. (2014) evaluated the effectiveness of intermittent superficial cooling (i.e., 5-min of wearing a cooling vest) in 10 college students over 60-min of intense American football training in TropC. Thermal sensation was significantly decreased by this technique but not thirst, RPE, nor heat illness symptoms.

Regarding the impact of cooling strategies on cognitive abilities, Lee et al. (2014) evaluated the efficacy of neck cooling on cognitive task (i.e., symbol digit matching, search and memory, digit span, choice reaction time and psychomotor vigilance test), following exercise-induced hyperthermia. They showed that the neck-cooling collar seemed to enhance performance only in the high-complexity tasks (i.e., search and memory test). Ando et al. (2015) also examined the influence of neck-cooling interventions (with a wet towel and fanning) on cognitive functions during an exercise in TropC (35°C and 70% rH). They particularly focused on working memory and executive function in eight participants at rest and after cycling 10-min in both conditions (i.e., TropC vs. TropC+cooling intervention) but found that the cooling intervention in TropC did not effectively attenuate the impairment of either. Bandelow et al. (2010) used another cooling method to examine the cognitive effects of exercising (i.e., complex visuo-motor, fine motor speed, visual/auditory and visuo-spatial working memories) in TropC in a series of three matches between two soccer teams. The environmental conditions were about 34°C and 63% rH. The players sat under a canopy in 25°C temperature for 15-min before the match and for 10-min during the half-time break. Of the four cognitive functions assessed, only complex visuo-motor speed was improved.

Thus, some cooling interventions may not be efficient enough (e.g., Shibasaki et al., 2017) or may even be counterproductive. Indeed, internal cooling by ingesting large volumes of cold water or ice during exercise might cause gastrointestinal discomfort, allergy or cold-stimulus headache in some individuals (Stevens et al., 2013). Given the disadvantages of certain conventional interventions and the complexity of implementing them during sports competitions, other less invasive and easier to implement techniques should be considered to avoid the inconvenience of overly restrictive or embarrassing cooling interventions and yet maintain the psychological benefits.

## Impacts of Alternative Strategies to Deal With Thermal Stress

In addition to the conventional interventions to deal with heat stress, other techniques can counter heat and TropC. Thus, these techniques may be used as alternatives or complements.

### Menthol Ingestion Techniques to Create a Cooling Sensation

A series of studies has dealt with subjective body cooling through menthol. Although menthol does not lower the core or skin temperature of athletes (Barwood et al., 2015), it stimulates the cold receptors (Cheung, 2010) and induces a cool feeling (Mündel and Jones, 2010), which modifies thermal perceptions. Menthol-induced cold perception has been shown to increase exercise intensity and performance in hot climate (see Cheung, 2010; Mündel and Jones, 2010) and in TropC (e.g., Riera et al., 2014). For example, Stevens et al. (2016) examined the impacts of ice-slurry ingestion and menthol mouth rinse (25 mL of an L-menthol solution at 22°C at a concentration of 0.01% during 5 s) prior to an endurance running performance in the heat. Although ice-slurry reduced the core temperature, it neither decreased the thermal sensation during exercise nor improved the performance on a 5-km run. In contrast, the menthol mouth rinse improved the thermal sensation during exercise and the running performance. However, care is needed when using this technique. Indeed, a false thermal afferent signal, as when the actual temperature of the skin differs from the cold perception, can increase the risk of developing heat-related illnesses (see Valente et al., 2015), the brain being fooled by the menthol-induced cold signal. Moreover, a high menthol concentration could alter thermal perception to an extent that a hot deep body temperature could be ignored. This could lead to even more serious health risks such as heat stroke or hyperthermia, and subsequently to generated cerebral blood flow reduction, brain temperature increase or/and cerebral oxygenation compromising (Bain et al., 2015).

Although these results suggest that the modified perceptive signal is stronger than the physiological signal, it should be possible to find a technique that retains this perceptual advantage without the risk of a side effect on health. Other techniques, such as mental techniques, might avoid the downside of a menthol intervention while maintaining its psychological benefits for motivation and performance. These can be used to manage thermal stress and create a cold feeling that could help athletes to psychologically deal with the thermal environment.

### Mental Techniques to Manage Thermal Stress

The techniques of mental preparation are useful to manage stress during competition, but it might be worthwhile to examine the classic mental preparation techniques to determine which ones would be most appropriate for coping with climate stressors. Many variables have been explored, including athletes’ resilience and adaptation (Fletcher and Sarkar, 2012). The question remaining: Which strategy is best for coping with climatic factors? In the field of sport and exercise, few studies have investigated psychological techniques in relation to ambient temperature. Barwood et al. (2008) tested whether a training package of four psychological skills (i.e., goal setting, arousal regulation, mental imagery, positive self-talk) would increase the distance covered during three maximal-effort runs of 90-min in the heat (30°C and 40% rH). They showed that the package lowered the temptation to reduce exercise intensity during the maximal-effort runs while increasing the distance covered by 8% in the last 90-min run. However, it was not possible to determine which of the skills had the beneficial influence. Wallace et al. (2016), therefore, used only one psychological skill (i.e., motivational self-talk) and showed that a 2-week intervention significantly improved endurance capacity and executive function in the heat. Similarly, it might also be interesting to examine how conventional mental interventions could be specifically adapted to coping with TropC. From this viewpoint and even though it has not been shown thus far, mindfulness might be an appropriate strategy. This technique has three components (*awareness* of current thoughts, emotions and bodily sensations; *acceptance*, which is a non-judgmental attitude toward one’s current thoughts, emotions, and bodily sensations; and *commitment* to goal-relevant attention focus and behavior) (Gardner and Moore, 2007; Thienot et al., 2014). Although the study did not take place in a particular climate condition, Haase et al. (2015) showed that a 7-week mindfulness intervention (2 full days+6 sessions of 90-min/week) changed the way high-level athletes treated interoceptive information and increased their ability to regulate the anxiety related to unpleasant feelings. This technique seems a more promising way to mentally prepare athletes for the Olympic and Paralympic Games in TropC.

### Mental Techniques to Create a Cold Feeling

Few studies in the health field have examined the effect of mental techniques such as hypnosis on thermal factors. Langlade et al. (2002) investigated the influence of hypnotic suggestion and showed that heat detection and heat-pain thresholds were increased. Younus et al. (2003) studied the effect of hypnosis (4 × 1-h/week) on hot flashes in ten healthy volunteers and four breast cancer patients and showed that the frequency, duration, and severity of the hot flashes were significantly reduced. Elkins et al. (2013) obtained equivalent results. However, another question is whether this technique is also effective for coping with the discomfort of a hot and wet environment. For this reason, it would be interesting to examine whether an intervention in TropC consisting of hypnotic cold suggestion would have specific effects on psychological markers (i.e., thermal comfort, thermal sensation, affect) and the motivation to perform exercise compared with a control situation (in TropC with a neutral intervention). Finally, the scientific literature has not yet studied the effects of TropC and cooling strategies on other psychological factors like motivation, self-confidence or flow states. It would therefore be interesting to examine the effects on these psychological factors that are directly involved in performance. Likewise, no studies have compared high performance athletes with mid-level athletes in terms of their ability to cope with heat stress. Thus, it would be interesting to examine whether psychological components such as motivation, resilience or achievement goals allow high performance athletes to feel perceptions (e.g., thermal comfort, RPE) more bearable than mid-level athletes.

## Conclusion

Although the conventional strategies are of interest for managing physiological responses during long-duration exercise (see Bongers et al., 2017), they have more relative effects on psychological factors, particularly in TropC. In addition to the disadvantages of implementing pre-cooling (cold-water immersion, cooling garments, cold packs applied to the head) or per-cooling (cold-water, ice-slurry, and/or menthol ingestion), there are also the psychological costs. Indeed, if the physiological benefits (central temperature decrement) are less than the psychological cost (increase in cognitive disturbance), the strategy in real performance situations will prove counterproductive particularly in highly demanding cognitive tasks.

The results concerning the efficacy of these techniques on cognitive abilities depend of the intervention used and the factor investigated. It seems very important to match the expected benefits of a particular technique (conventional or alternative) with the type of performance. Although all psychological factors are important in high-level practice, psychological needs differ across sports. On the one hand, precision sports (e.g., 50-m pistol, archery), team sports (e.g., football, basketball) and duel sports (e.g., tennis, fencing) will primarily require cognitive resources (e.g., attention, decision-making) through subjective intervention (e.g., cold suggestion), and the use of objective strategies (e.g., a cooling vest during timeouts) could be beneficial if they do not disturb the athlete. On the other hand, aerobic sports (e.g., the marathon, 50-km race walk) will mainly require motivational resources through training adaptation in TropC, the use of objective strategies (e.g., cooling equipment before the race), and the use of mental techniques (e.g., mindfulness intervention) should be beneficial.

Last, a parameter that deserves more study is inter-individual variability. Some high-level athletes may be much more supportive of certain strategies, whereas, others prefer one or a combination of different strategies. Not all athletes are inclined to drink crushed ice or take an ice bath before a race. Cooling techniques are not necessarily adapted to all sports modalities and all athletes. We need to focus on the strategy (not the method), which will depend on the severity of the environment and the athletes’ perceptions. As perceptions differ from one athlete to another, the psychological feelings are essential. This aspect is enhanced by the fact that the majority of the studies presented in this review are with weak samples and with mid-level athletes as there are few studies of high-level athletes related to the climate. For this reason, we encourage delegations to have their athletes train in TropC a few weeks before the Tokyo Games (i.e., acclimation training during preparation camp) and to arrive on site early enough to have the time to test strategies and find the most effective one in terms of perceptions (e.g., thermal comfort, RPE), cognitive abilities (e.g., reaction time, attention) and feelings and emotions (e.g., self-confidence, motivation or flow states). With this goal in mind, we also encourage delegations to consider easy-to-use alternative strategies to preserve the psychological benefits in TropC without constraining the athletes to cooling interventions that are potentially deleterious for their performance (see Table 1).

**TABLE 1:** Practical implications for dealing with thermal stress.

1	Consider acclimatizing athletes by conducting training sessions for the Olympic preparation period in a tropical environment (training base camp in Japan or a region with a TropC, like the French West Indies). By becoming psychologically accustomed to the difficult conditions, the athletes will experience less stress (heat stress) and disturbance once in Japan. This will also allow for experimentation and selection of pre-cooling methods for all types of outdoor exercise (not only aerobic exercise). To facilitate acclimatization and not just jet lag and chronobiology issues, plan to arrive in Japan as soon as possible before the start of competitions. Given the very special conditions of the Tokyo City (i.e., UHI), consider setting up and training at the nearest competition venues.

2	Anticipate the physical preparation before acclimatization in Tokyo. Schedule physical training in the warmest hours of the country of origin, in warm rooms and with windproof clothing. Once you are in TropC in Japan, keep the same physical goals before setting higher goals. Then, increase the goals more gradually than if you were in a temperate climate. You can begin to get used to gradually by scheduling a physical training at the coolest hours of the day (early in the morning and evening), then after 1 week scheduling training hours on the same time slots of the competitions. Recommendations for nutrition, hydration and sleep, important in a temperate climate, are even more numerous in TropC (e.g., drink fresh water regularly before, during and after exercise, nap and sleep in cool rooms to facilitate better recovery).

3	Consider all conventional cooling techniques that do not seem to have an adverse effect on psychological factors. Consider a set of pre-, per-, post-, and combined cooling interventions in agreement with each athlete. For example, drinking cold water at the preferential temperature, cold wet towels on the head, and a cold jacket before and after warm-up and during timeout of the competition. Check the rules of the sport to guard against any appeal from the organizers or opponents concerning the use of a cooling strategy (e.g., cold packs, collar, cooling vest) during competition.

4	With each athlete, try to find the most effective and enjoyable technique or combination of techniques. Although this has not been demonstrated, it is possible that there is a strong inter-individual difference in the acceptability and effectiveness of the same technique across athletes. Some techniques may, for example, be psychologically beneficial for some athletes, neutral for others, and disturbing for others.

5	While it is important to support and encourage the athlete in their efforts, it is even more important to do so in TropC where physical goals are more difficult to achieve. With each athlete, select from among all the mental preparation techniques those that seem most adapted to cope with TropC (e.g., the mindfulness program). Include the techniques selected for the Olympic or Paralympic Games within the athletes’ performance routines so that they can obtain the benefits (i.e., better thermal comfort, reduced perception of exercise difficulty, increased motivation) without suffering undesirable effects (i.e., concentration decrement, distraction, discomfort).

6	Use performance routines (including selected cooling and mental interventions) at each training session prior to the Games to allow athletes to obtain and maintain a flow state for longer times during competition.

## Author Contributions

All authors contributed to the manuscript redaction, from the plan conception to the review of literature to the corrections.

## Conflict of Interest Statement

The authors declare that the research was conducted in the absence of any commercial or financial relationships that could be construed as a potential conflict of interest.
